# A New Source of Electricity

**Published:** 1881-08

**Authors:** 


### EDITORIAL, ETC.

*A New Source of Electricity.*—It was made known many years ago by Joule that when a bar of iron is inserted into a coil of wire through which a current of electricity is passing, the bar is appreciably lengthened. That, within certain limits, a physical effect differs from its cause only in the order of time, has also been long known. If, for example, electricity produces heat, heat will produce electricity; if magnetism can produce electricity, electricity can produce magnetism. If chemical actions are attended with light, light under suitable circumstances may be expected to produce chemical actions It was a ready inference, therefore, from Joule's experiment, that if a bar of iron within a coil of wire were elongated by the application of mechanical means, it ought to produce electricity. Prof. E. A. Dolbear, in 1828, while engaged in measurements of the amount of elongation experienced by bars submitted to the action of electricity, was the first to think of testing the inverse of Joule's phenomenon—the possibility of producing electricity by forcible elongation. The result answered his expectation. A rod was passed through a coil connected with a galvanometer, and then the rod was put under tension in a testing machine. As the strain was put on the galvanometer was violently affected, and this was continued as the stretching of the rod continued. When removed the bar was found to be strongly magnetic and hot. Under compression similar effects were produced. It was found that metals other than iron or steel give but feeble results from stress. From Dolbear's experiments we may infer that a machine in which rods encircled with coils are hammered rapidly on their ends is destined to be constructed at an early day for the production of currents of electricity. Whether they will prove economically profitable remains to be seen.

